# Supplementary material for: Are carbon emissions trading and green financial instruments synergistic? -Comprehensive quantitative research based on content analysis
Source: PLoS One. 2024 Mar 7;19(3):e0298601. doi: 10.1371/journal.pone.0298601 (PMC10919601; doi:10.1371/journal.pone.0298601)
Supplement: S1 Data — (ZIP) [file pone.0298601.s001.zip › Data/地级市Z_F工作报告.zip!/░▓╗╒/┬φ░░╔╜╩╨/└┤╫╘╣1⁄2╓┌║┼í╛┬φ┐╦╩2╛▌═°í┐_2007.docx]

# **政府工作报告（2007）**

【字体大小：[大](http://zwgk.mas.gov.cn/openness/detail/content/javascript:doZoom(18)) [中](http://zwgk.mas.gov.cn/openness/detail/content/javascript:doZoom(15)) [小](http://zwgk.mas.gov.cn/openness/detail/content/javascript:doZoom(14))】

                                                                        市长 姚玉舟
                                                                       (2007-01-15)
各位代表： 

    现在，我代表市人民政府向大会作工作报告，请予审议，并请市政协委员和其他列席人员提出意见。 
    一、2006年政府主要工作 
    刚刚过去的2006年，是马鞍山历史上很不平凡、具有特殊意义的一年。这一年，我们全力实施“十一五”规划，实现了又好又快发展的崭新开局；这一年，我们认真落实市第七次党代会提出的新任务，加快了在全省率先建成全面小康社会的步伐；这一年，我们隆重庆祝建市五十周年，更加激发了全市人民建设美好家园的雄心壮志；这一年，我们积极主动地贯彻国家宏观调控政策，经济发展的速度、质量、效益均好于预期，开创了马钢、开发区和县区等各经济板块竞相发展的新局面。一年来，在省委、省政府和市委的坚强领导下，我们全面贯彻党的十六届五中、六中全会和省第八次党代会、市第七次党代会精神，坚持以科学发展观为指导，抢抓中部崛起、东向发展新机遇，强力推进招商引资、全民创业等各项重点工作，圆满完成市十三届人大五次会议确定的目标任务，在马鞍山发展史册上谱写了率先发展、科学发展、和谐发展的新篇章。 
    （一）经济持续快速健康运行，率先发展的态势进一步显现。全市实现生产总值突破400亿元，达415亿元，增长16%；人均生产总值迈上4000美元新台阶。经济增长方式加快转变，万元GDP能耗2.44吨标准煤，下降4.7%。实现财政收入74亿元，增长16%，其中当涂县财政收入增幅超过40%，马鞍山经济开发区财政收入突破3亿元，三个区财政收入均超2亿元。完成全社会固定资产投资260亿元，增长35.9%。三次产业均呈加速发展之势。中小企业异军突起，全年新增规模以上工业企业101户，规模以?新技术产业产值160亿元，增长22%。在前两年的基础上再次出台落实支农惠农政策措施，促进农村经济稳步发展。全年实现农业增加值18.5亿元，增长6.8%。第三产业加快发展。全年社会消费品零售总额73.5亿元，增长14.3%。旅游业总收入14.8亿元，增长22.1%。金融机构年末本外币存款余额373.1亿元，贷款余额268.5亿元，分别比年初增加54.9亿元和73.2亿元，支持地方经济发展的力度进一步加大。总体上看，主要经济指标人均值继续保持全省领先，与长三角发达城市的差距进一步缩小。 
    （二）招商引资在创新中加力推进，对外开放水平全面提升。积极创新招商方式，全市组建100个招商小分队进驻沿海和经济发达地区，建立了覆盖广泛的招商网络。精心组织参加香港徽商大会、首届中博会、上海台商大会、南京重洽会等大型招商活动，取得一批重要招商成果。狠抓招商引资责任落实，吸引广州立白、家乐福等大项目落户，促成圣戈班、中橡等一批外来投资企业增资扩股。全年实际利用外资2.05亿美元，实际利用内资100亿元，分别增长40.1%和120.5%。马鞍山经济开发区实际利用外资1.5亿美元，各项经济指标继续位居省级开发区前列。慈湖经济开发区基础设施日趋完善，区内完成固定资产投资突破20亿元；工业销售收入达到46亿元，增长64.1%；实际利用外资3384万美元，增长65%。当涂经济开发区发展框架进一步拉开，各项经济指标快速增长。三个区和当涂县的各类工业集中区都获得长足发展。积极实施“走出去”战略，完成外贸进出口总额20亿美元，增长62.5%。其中出口5亿美元，增长59.2%，机电、高新技术产品出口均实现成倍增长。外经营业额增长15%，外运量增长3倍，境外劳务输出356人，常年来马帮助工作、从事技术服务的境外专家多达百人。与国内外友好城市交往不断增多。区域经济合作取得新进展。加快了与长三角城市对接的步伐，与皖江城市的协作进一步加强。成功承办中部崛起与安徽发展、南京都市圈发展等论坛。马钢与宝钢签订了战略联盟框架协议；成功重组合钢主业，马钢合肥公司年底扭亏为盈的目标提前3个月实现。开放环境进一步改善，我市荣列国家统计局发布的中国投资环境百佳城市榜。 
    （三）重点项目建设快速推进，经济增长后劲持续增强。认真贯彻中央宏观调控政策，按照国家产业政策的要求，加大力度推进重点项目建设，连续获得省“861”行动计划考评第一名。马钢新区建设创造了“马钢新速度”，铁前系统基本具备投产条件，钢轧系统进入设备单体调试阶段；车轮扩能改造等项目建成发挥效益，高村二期、白象山等矿山建设正在积极推进。万能达二期、中橡扩能二期、蒙牛乳酸生产线等17个重点项目建成投产，山鹰30万吨纸板技改、圣戈班球墨铸铁管扩能等一批项目扎实推进，马鞍山长江大桥试桩动工，雨润肉制品、沃尔玛购物中心等21个项目按期开工。坚持合法利用土地，严格履行用地报批手续，促进了重点项目的实施。全年共完成重点项目投资136亿元，其中列入省“861”行动计划项目投资113.7亿元，投资量继续处于全省前列。结合制定“十一五”发展规划，编制储备了一批新型工业化、新农村建设、服务业和社会事业类重大项目。突出抓好重大项目前期工作，大唐电厂、联合电厂、城际铁路等项目取得重要进展。 
    （四）全民创业蓬勃兴起，民营经济加速发展。深入推进东向发展、全民创业，扎实开展“企业服务年”活动。出台落实支持全民创业的30条政策措施，降低创业门槛，培育创业主体，创新帮扶机制，全民创业环境进一步优化。依托各类工业集中区，建成各类创业基地10万多平方米，创业平台日趋完善。在区县设立创业服务中心，开展政策咨询，加强创业指导。采取多种形式促进银政企合作，新组建担保公司6户；银行业积极创新金融产品和服务，加大对企业的贷款，中小企业和创业者的融资难问题得到缓解。大力扶持民营企业扩大规模、二次创业，推动民营经济发展迈上新台阶。全年新发展个体工商户5820户，新发展私营企业1524家，民营经济占全市经济总量的比重提高2.1个百分点。全市初步形成了百姓创家业、能人创企业、干农村建设扎实推进，“三农”工作取得显著成效。制定了推进新农村建设的若干政策措施，编制了新农村建设总体规划、产业发展规划、村庄布点规划和各专项配套规划，修编了乡镇总体规划。市级“一镇十村”新农村建设试点进展顺利，当涂县被确定为中央财政整合资金支持新农村建设试点县。农业生产战胜秋旱、病虫害等多种灾害，实现粮食总产40.2万吨，好于上年水平。农业优质化水平持续提升，2户企业获得无公害农产品产地认定，8个产品获得绿色食品认证。农业区域化生产、规模化经营取得新进展。苗木花卉基地建成面积5600亩；速生丰产林基地发展到1.8万亩；奶牛养殖基地建成投产，养殖奶牛近1万头，蒙牛现代牧场带动效应开始显现；水产生态养殖基地建成1700亩，辐射带动全市水产生态养殖面积20万亩；100亩以上的粮食规模种植户近500户。农业产业化加快发展，新增省级龙头企业4户，新建农民专业合作社8家。大力发展“三资”农业，全年到位资金4.5亿元。“万村千乡”市场工程建设试点扩大到三区一县，建成乡级店38个、村级店255个，农村消费品市场日趋完善。农村基础设施建设加快推进。围屏至乌溪公路竣工通车，全年建成“村村通”公路310公里。乌溪排灌站建成使用，水利工程抗旱效益得到充分发挥。城市供水管网向农村延伸，大公圩中心水厂开工建设，农村安全饮水工程受益人口已达43万。小城镇建设完成投资10.5亿元，承载功能明显增强。农村社会事业快速发展。乡镇卫生院的医疗条件进一步改善，6个省级农村文化站示范点启动建设，当涂县农村远程教育项目全面建成，20户以上自然村“村村通”广播电视工程顺利实施。第二次全国农业普查工作全面启动。 
    （六）城市建设管理力度加大，市容市貌发生明显改观。完成《马鞍山市近期建设规划（2006—2010）》等规划的编制，《采石风景区总体规划》通过评审，规划龙头作用进一步发挥。基础设施建设加快推进。天门大道北段、葛羊路东段等道路建成通车，新建、改造城市道路19公里。公用设施日趋完善。500千伏滁马输变电、220千伏恒兴变启动送电，移动、电信枢纽项目相继开工。向山垃圾场生活垃圾无害化处理达到国家一级标准，王家山污水处理厂改扩建工程按进度推进，天然气供气范围向农村延伸，市区至当涂县城公交车正式开通。房地产业稳健发展，全年竣工房屋面积235万平方米，整治老旧住宅小区9个，城乡居民住房条件进一步改善。天天、湖西路等集贸市场建成开业，重阳路、团结西路等一批市场开工建设，全市商业网点进一步完善。住房公积金归集额、贷款发放额分别增长16.2%和39.9%，当涂县公积金管理与市区接轨。以荣获“国家环保模范城市”为动力，全面启动生态市建设。城市中心区绿化美化亮化全面完成，采石河节庆广场基本完工，雨山湖水域生态治理扎实推进。深入开展违法排污整治专项行动，强化污染控制，有效保护了居民生活环境。扎实开展“城市管理年”活动。强力推进违法建设拆除工作，全年共拆除城区违法建设60多万平方米。开展散装货车密闭改装和渣土运输抛洒整治，整治占道经营、户外广告、主干道门头招牌，合理设立摊群点，市容环境卫生状况得到改观。集中整治城区“四小车辆”，规范了营运市场秩序，营造了安全有序的交通环境。积极探索在市容环卫领域实行网格化管理，推动城市管理逐步向基层延伸，城市管理长效机制初步建立。 
    （七）重点领域改革加快实施，体制机制增添新的活力。巩固扩大企业改革成果，中小企业改革扫尾攻坚取得重要进展，已改制企业不断焕发出新的生机和活力。马钢、星马、山鹰3家上市公司股权分置改革顺利完成。市属3户资产运营公司重组工作基本完成。马钢在A股市场首家公开发行55亿元分离交易可转换公司债券。中钢天源公司在深圳交易所成功上市，募集资金1.3亿元。十七冶完成整体改制，成为产权多元化大型施工企业。交通银行马鞍山分行获中国银监会批准筹建。认真落实国有资产经营责任制，强化国有资产监管，国有资产实现了保值增值。深化行政管理体制改革，取消和调整各类审批事项57项；将涉及16个政府部门的27项事权下放到区，进一步强化了区级功能。生产经营类事业单位改革取得阶段性成果。市文化、卫生系统事业单位人事制度改革试点积极推进。水管体制、港口体制改革开始实施。动物疫病防控长效机制初步建立。当涂县农村综合改革试点基本完成，农村发展活力进一步增强。 
    （八）科技教育和社会事业全面进步，精神文明创建活动扎实开展。召开了全市科技大会，确立了建设创新型城市的战略目标，制定实施一系列政策措施，着力增强科技创新能力。国家“863”新材料基地建设取得重要进展，科创中心被批准为国家级创业中心，环保科技园建成使用，留学人员马鞍山创业园获准设立。国家金属矿产资源高效循环利用工程研究中心开工建设，国家金属矿山固体废弃物处理与处置工程技术研究中心通过科技部验收。产学研合作签订项目百余项，专利申请量达180件。大力实施质量兴市和品牌战略，继马钢H型钢首获中国名牌后，海狮牌毛巾又被授予中国名牌产品称号，另有7个产品获国家免检产品称号，3个产品进入省名牌行列。方圆回转支承股份有限公司等5家企业被认定为国家火炬计划重点高新技术企业，惊天液压机械制造公司等46家企业被认定为省级高新技术企业。当涂县进入国家科技富民强县专项行动计划行列，花山区工业集中区被批准为省级民营科技园。各类教育持续协调发展。素质教育全面实施，“普九”成果得到巩固，基础教育继续向均衡方向发展。普通高考成绩创历史新高。为新建小区配套的师苑小学、金瑞初中等中小学建成使用。安工大附中、二十二中跻身省示范高中行列。职教骨干专业实训基地加快建设，全市职普比接近1:1。河海大学闻天学院（筹）、安工大东校区二期启动建设，安工大职业技术学院迁建加快推进。文化事业日趋繁荣。成功举办首届国际太极拳名家论坛、中国李白诗歌节，李白墓园列为国家重点文物保护单位，当涂民歌入选首批国家级非物质文化遗产名录。爱国卫生运动深入开展，顺利通过国家卫生城市检查复审。医疗设施建设扎实推进，市传染病医院一期工程建成交付使用。重大疾病预防控制工作进一步加强，医疗服务质量有新的提高。社区卫生服务全面推开，雨山区被评为“全国社区卫生服务示范区”。体育事业迈上新台阶。马鞍山籍运动员李臻夺得第15届亚运会男子双人皮艇1000米冠军和500米铜牌，我市运动员参加省十一届运动会取得团体第三的历史最好成绩，金家庄区荣获“全国全民健身周先进单位”称号。狠抓农村流出人口计生管理薄弱环节，开展城区流动人口计生集中服务，加强“两非”案件查处工作，全年人口出生率控制在11‰。民族宗教工作进一步加强。其他各项社会事业都有新的进步。 
    精神文明建设深入推进。以建市50周年为契机，组织开展了系列庆祝活动，对广大群众进行广泛深入的爱国主义、集体主义、社会主义教育。大力开展社会主义荣辱观教育，促进公民道德建设。和谐社区建设取得明显成效，花山区东苑社区被授予“全国文化先进社区”，安工大社区被评为“全国社区侨务工作先进集体”。马钢荣获全国国有企业创建“四好”领导班子先进集体称号。围绕“平安马鞍山”建设，积极构建社会治安动态防控体系，加强群体性事件预防和处置，依法打击各类刑事犯罪活动，刑事案件发案率进一步下降??带案下访和包案制度，认真排查调处信访隐患，使社会矛盾和不稳定因素得到有效化解。“五五”普法全面启动，依法治市工作取得新进展。基层民主政治建设不断加强。驻马部队和后备力量建设取得新成绩，创建双拥模范城市工作扎实开展。国防指挥中心建成使用，军地共建的澄釜路竣工通车。成功组织了马和轮渡渡场开设演练，民兵遂行任务能力得到提升。双拥优抚安置政策有效落实，随军家属生活补贴标准由100元提高到240元。人民防空基础设施建设力度不断加大，城市防空抗毁能力进一步增强。 
    （九）就业和社会保障体系不断完善，人民生活水平进一步提升。城乡居民收入持续增长，城市居民人均可支配收入13700元，农民人均纯收入5200元，分别增长14.8%和15.3%，恩格尔系数下降到38.1。城乡就业状况继续改善。完善了积极的就业再就业政策措施，突出抓好就业和创业培训，统筹推进城乡劳动者就业。全市城镇实现新增就业2.8万人，其中下岗失业人员再就业1.5万人，“4050”人员等困难群体再就业2700人，城镇登记失业率为3.7%。在全国首创村级劳务公司，全市农村输出劳动力由14.8万人增至17万人。完成就业再就业技能培训1万人，创业培训800人，培养新技师380人。稳步推进劳动合同和集体合同制度，认真解决涉及农民工利益问题。社会保障制度进一步健全。全市基本养老、失业、医疗保险综合覆盖率达98%，工伤、生育保险覆盖面继续扩大。全民关注的城镇非职工居民基本医疗保险制度在全省率先实施，当年参保率达91.2%，有效保障了城镇非职工居民的大病、重病医疗；新型农村合作医疗保障制度得到巩固和发展，城乡贫困群众医疗救助基金已经建立，医疗社会保障网基本实现全覆盖。被征地农民养老保障制度平稳运行，参保人数达4.4万人。农村低保制度覆盖13个乡镇，覆盖面扩大到75%。上调了企业退休人员养老金、劳动者最低工资标准、出中心退养人员基本生活费、失业人员失业保险金、城市低保标准以及社区干部生活补贴，低收入者收入水平进一步提升。农村敬老院改扩建力度加大，农村五保供养工作得到加强。“慈善一日捐”活动有效发挥了济困助贫的作用，社会救助体系框架基本建成。全年被征地农民安置房建成交付使用4879套、已封顶9069套，建成廉租住房100套，廉租住房补贴标准进一步提高。经过不懈努力，市政府10件为民办实事项目全部按期完成，有效解决了人民群众关注的一批重难点问题。 
    （十）政府自身建设得到加强，行政效能有新的提高。以提高工作效能、优化发展环境为主题，在政府系统集中开展加强效能建设活动，取得明显成效。出台《关于进一步提高行政领导工作效能的若干规定》，实行政府部门行政首长问责制，全面推行首问负责制、一次性告知制等六项制度。对部分审批流程和公共服务流程进行再造，实施“授权委托审批”制度，全面清理行政事业性收费，向社会公布政府部门法定职责，进一步规范行政行为。认真办好“12345”市长公开电话和“政风行风热线”，开辟市政府网站在线交流栏目，进一步畅通与市民沟通交流渠道。出台实施《突发公共事件总体应急预案》、20个专项预案和59个部门及县区预案，政府应急管理机制逐步健全。一年来，市政府向人大报告工作、向政协通报情况制度得到全面落实，政府系统承办的9件人大代表议案、99件建议批评意见、6件市人大常委会审议意见、1件市人大主任会议意见和325件政协提案按要求办复。对市人大、政协调查社情民意反映的重要民生问题，及时予以办理。建立市政府负责同志领衔办理人大代表议案、建议、批评、意见和政协提案制度，市政府负责同志先后20多次约请、走访市人大代表和市政协委员，广泛吸纳基层群众参政议政，建言献策渠道进一步拓宽。加强与各民主党派、工商联及人民团体的联系，注重发挥舆论和社会的监督作用。开展商业贿赂专项治理，强化领导干部任期经济责任审计和政府投资建设项目审计，加强重点项目专项执法监察，及时纠正了损害群众利益的不正之风。认真贯彻惩治和预防腐败体系建设实施纲要，狠抓廉洁自律各项规定的落实，促进了政府系统廉政建设。 
    各位代表！过去的一年，我们在新的起点上迈出了新步伐，取得了新成绩。这是省委、省政府和市委正确领导的结果，是全市人民团结拼搏的结果。在此，我代表市人民政府，向辛勤工作在全市各条战线上的广大干部群众，向给予政府工作热情支持和有力监督的全体人大代表、政协委员，向各民主党派、工商联、无党派人士、各人民团体和广大离退休老同志，向中央和省驻马单位以及驻马解放军、武警官兵和政法干警，向在我市创业的境内外投资者、建设者和海内外友好人士，表示衷心的感谢并致以崇高的敬意！ 
    我们也清醒地看到，前进的道路上还面临不少矛盾和问题。主要表现在：一是总体发展水平与长三角先进城市相比仍有较大差距，在省内保持领先、实现率先发展的压力不断增大。二是经济运行的结构性、素质性矛盾仍较突出，市场约束、资源要素供给和环境保护的压力持续加大，转变经济增长方式的任务十分艰巨。三是经济社会统筹发展的长效机制尚未建立，城市建设管理等方面还存在薄弱环节，构建和谐马鞍山任重道远。四是政府职能转变还难以完全适应新形势新任务的要求，服务效能和队伍素质有待进一步提高。这些矛盾和问题，我们将在今后的工作中努力加以克服和解决。 
    二、2007年发展目标及主要经济工作 
    2007年是全面落实市第七次党代会精神、率先建成全面小康社会的重要一年，也是本届政府任期最后一年，我们肩负着光荣而艰巨的使命。综观当前国内外形势，我们仍然面临着机遇与挑战并存、机遇大于挑战的发展环境。经济全球化和区域经济一体化深入发展，国际生产要素流动和产业梯度转移加快，为我们优化经济结构、促进产业升级带来新的空间。随着WTO过渡期结束，服务业等领域将进一步对外开放，有利于我们加快开放步伐，提高外向型经济发展水平。我国城市化加速、新农村建设加快和消费结构升级所带来的巨大需求，将使工业化、市场化基础较好的马鞍山从中获得新的发展动力。中央促进中部地区崛起的战略已经进入全面实施阶段，省委、省政府把马芜铜宜城市群作为东向发展的第一方阵，使我市独特的区位交通优势、丰富的资源优势和良好的产业优势更加凸显。经过多年坚持不懈地加大有效投入，一批重大项目陆续建成，相继进入投资回报期，我市加快发展的动力和后劲明显增强。特别是率先建成全面小康社会的历史新任务，必将更加激发全市人民建设马鞍山的主人翁责任感。同时也要看到，我市正处于工业化转型、城市化加速、市场化完善、国际化提升的时期，面临着经济增长成本上升、资源环境约束增大、区域竞争日益加剧的严峻挑战。在贯彻执行国家宏观调控政策、实施增值税转型试点的过程中，也会遇到一些暂时的困难和问题。因此，我们一定要增强机遇意识、责任意识和忧患意识，准确把握国际、国内发展大势，因势利导、顺势而为、乘势而上，奋力开创率先发展、科学发展、和谐发展的新局面。 
    今年政府工作的总体要求是：以邓小平理论和“三个代表”重要思想为指导，以科学发展观统揽全局，认真贯彻党的十六届五中、六中全会、中央经济工作会议和省第八次党代会精神，全面落实市第七次党代会的各项部署，坚持东向发展和全民创业，着力推进自主创新和改革开放，加快调整经济结构和转变增长方式，切实加强资源节约和环境保护，促进社会发展和解决民生问题，保持和扩大经济社会又好又快的发展势头，确保全面和超额完成本届政府的各项目标任务，为率先建成全面小康社会、加速构建和谐马鞍山奠定坚实基础。 
    按照这一总体要求，我们立足于主要经济指标人均值继续保持全省领先、总量在全省争先进位、增幅位居长三角前列，进一步缩小与长三角先进地区的差距，对全年任务进行安排。今年的主要预期目标为：生产总值增长13%以上，财政收入增长12.5%，全社会固定资产投资增长15%以上，实际利用外资2.5亿美元，实际利用内资110亿元，外贸进出口总额完成15亿美元，社会消费品零售总额增长14%，城市居民人均可支配收入、农民人均纯收入分别增长10%以上，单位生产总值能耗下降3.4%左右，主要污染排放量下降2%，城镇登记失业率控制在4%以内，人口出生率控制在省下达的指标范围内，居民消费价格指数控制在3%以下。实际工作中，要争取完成得更好更快更全面。为此，将重点做好以下几方面工作。 
    （一）在更高层次上推进开放改革，加速经济国际化、市场化步伐 
    加强和改进招商引资工作。强化“走出去”与“请进来”工作力度，增强重点招商活动的实效性，实现驻点招商新突破。进一步优化投资服务环境，健全重大招商项目快速反应和综合协调服务机制，狠抓招商引资工作落实。整合涉外部门招商资源，加强高素质、专业化招商队伍建设，调动社会力量招商积极性，进一步扩大招商引资成果。充分发挥企业主体作用，突出抓好以商招商和产业招商，实现招商引资的良性循环。更加注重引进牵动力强、带动性大的重大项目和高新技术产业，引导外资进入高效农业、现代服务业领域，突破社会事业利用外资的薄弱环节，加速形成宽领域利用外资新格局。 
    大力推进开发园区建设和发展。高起点、高标准加快各类开发园区建设，提高投资强度和??级开发区为动力，加速推进马鞍山经济开发区南区建设，完成“一纵三横”道路网和配套市政设施建设，促成一批优质项目尽快建成发挥效益，全年实际利用外资1.6亿美元、内资10亿元以上。进一步完善慈湖经济开发区基础设施体系，协调推进项目建设，着力增强自我积累、滚动发展的能力，全年实际利用外资4000万美元、内资14亿元以上。支持当涂经济开发区上规模、上质量，全年实际利用外资2000万美元、内资10亿元以上。推动三区工业集中区加快升级，超前谋划大桥经济，实现引资载体集聚的新突破。 
    积极扩大对外经贸和区域合作。扶持壮大外贸主体，支持重点企业扩大出口规模，鼓励关键设备、核心技术和重要原材料的进口。加快建设服装出口加工基地，增强机电、高新技术产品的国际市场竞争力，切实转变外贸增长方式。积极扩大对外经济技术交流与合作，推动外经企业开发利用境外资源、承揽对外援建项目、开展外包服务工作，发展对外运输和劳务输出。进一步加强与长三角地区的合作对接，全方位参与南京都市圈建设，依托东部发达地区加快经济国际化步伐。大力拓展与皖江城市的合作领域，共同打造马芜铜宜现代城市群，更好地担当全省东向发展排头兵的重任。 
    深化企事业单位改革。全面完成国有企业改革扫尾工作，支持马钢深化改革，健全现代企业制度。力争方圆回转支承股份有限公司成功上市。完善国有资产运营公司管理体制，建立国有资产经营者业绩考核体系，规范激励和约束机制。分类推进事业单位改革。全面完成生产经营类事业单位转企改制工作，逐步推进社会公益类事业单位改革，加快建立事业单位新型管理体制和运行机制。全面推进事业单位人事制度改革，积极推行公益类事业单位全员聘用合同制，开展事业单位岗位设置试点，完善专业技术人员分配激励机制。加大社会事业领域体制改革力度，建立健全与社会主义市场经济相适应的公共服务体系。 
    （二）大力促进工业结构优化升级，实现工业经济发展新突破 
    全力推动马钢发展大跨越。今年是马钢发展史上又一个具有里程碑意义的年份，新区500万吨钢工程将全面建成，标志着马钢向具有国际竞争力的现代化大型企业集团迈出新的一步。支持马钢加快新区设备安装最后冲刺和工程扫尾步伐，确保上半年全面建成投产，年内实现月达产。支持马钢继续抓好冷轧硅钢和车轮扩能改造二期等技改项目，加快推进矿山建设。帮助做好马钢外部运输保产、厂区周边治安环境整治等工作，为马钢全年钢产量突破1300万吨，销售收入突破400亿元创造良好环境。同时，支持马钢深入推进标准化工作，加快实施低成本战略和品牌战略，及早研究和实施“十一五”后期结构调整和技术改造方案，努力实现装备的大型化、现代化，持续提升企业竞争力。 
    集中力量实施一批重点工业项目。坚持把优化投资结构摆在首位，以落实省“861”行动计划为着力点，推动一批事关发展全局的重大项目建设，不断提升工业经济整体素质。快速推进山鹰30万吨纸板、雨润肉制品、圣戈班球墨铸铁管扩能、广州立白日用化工品等项目建设，力争年内投产发挥效益。支持蒙牛公司与法国达能公司开展合作，加快推进三期项目建设，不断扩大生产规模。鼓励星马专汽大力发展混凝土泵车生产，支持华菱重卡拓展国内外市场。努力创造条件，促进华菱公司与世界著名大公司合资合作。大力支持惊天液压机械制造公司机器人等高科技项目建设，提升企业核心竞争力。进一步落实重大项目储备机制，扎实推进大唐电厂、联合电厂等重大项目前期工作，争取早日获准建设。加强土地供应管理，优先保证重点项目建设用地。 
    促进中小企业快速发展。加大对重点企业经济运行的协调力度，着力化解企业发展中的制约因素。支持企业加快技术改造步伐，做大做强“专精特新”企业群体，培育一批对行业发展有示范带动作用的龙头企业。制定实施产业集群发展规划，提升磁性材料、刃模具机械加工等产业的集聚效应，延伸汽车及零部件、船舶、金属压延、纺织服装、农副产品深加工产业链，构筑具有较强实力和鲜明特色的产业集群。把淘汰落后产能作为调整优化经济结构的重要途径，依法关闭技术落后、能耗过高、污染严重的产能，不断提高发展的质量和效益。认真落实《安徽省中小企业促进条例》，强力推进“万千百十”工程，大力培植工业“小巨人”。全年力争规模以上工业增加值增长20%以上，新增规模以上工业企业超100户，新增销售收入过亿元企业10户以上。 
    （三）坚持城乡统筹发展，加快推进新农村建设 
    狠抓新农村建设规划的实施。以增加农民收入为核心，以调整农业结构为主线，以发展农业产业化为纽带，统筹推进新农村建设。切实增加投入，以实施全省“千村百镇”示范工程为抓手，落实好中央财政整合资金支持新农村建设试点县项目，推动银塘镇等国家农业综合开发项目建设，争取丹阳镇丹东村列入全国新农村建设试点村。推进农村公路建设，开工建设澄心至湖阳公路二期工程，在全省率先完成“村村通”工程建设任务。加强农田水利建设，重点实施大公圩排涝泵站工程，鼓励农民对直接受益的小型水利设施投工投劳，提高农业抗御灾害能力。全面完成农村安全饮水工程建设，实现农村集中居民点自来水供给率达到100%。加快农村污水和垃圾处理设施建设，改善农村生活环境和村容村貌。大力推进小城镇建设，促进中心镇加快发展。把发展农村经济作为新农村建设的重要支撑，培育壮大一批县乡骨干企业，做大做强县域工业经济。鼓励扶持农民创业，加快农村劳动力转移步伐，多渠道促进农民增收。实施农村户户通电话工程，大力推进有线电视“村村通”工程。加强对农民的宣传教育，充分发挥他们在新农村建设中的主体作用。 
    加快发展现代农业。围绕构筑现代农业产业体系，重点推进优势农产品基地建设，促进农业规模化发展、标准化生产。扩大农业订单面积，增强农业品牌意识，提高优质农产品覆盖率。坚持以产业化提升农业，着力壮大加工型龙头企业，力争新增年营销收入过5亿元企业1户。编制推出一批优势特色农业招商项目，引导“三资”投入农业综合开发，确保全年到位农业“三资”4亿元以上。支持供销社等各类机构发展农村合作经济组织，提高农业组织化、市场化水平。大力发展特色产业示范村，推动形成“一村一品”、“一乡一业”，打造规模块状农业。推进“农业科技进村入户”工程，促进农业科技成果转化和示范推广。全面贯彻《农产品质量安全法》，进一步健全质量标准、检验检测和质量认证体系，确保农产品质量安全。 
    扎实开展农村各项改革。继续深化农村综合改革，积极稳妥地开展化解乡村债务工作，巩固完善农村义务教育经费保障机制。在农民自愿的基础上，依法开展农村土地承包经营权流转试点，发展多种形式适度规模经营。继续实施兽医管理体制改革，不断完善兽医技术支持和畜牧技术推广体系。加快林业管理体制改革，探索国有林场改革办法，全面推行集体林权制度改革。稳步实施水管体制改革，进一步创新农村小微型水利工程产权制度和建设运营新机制。加快推进征地制度改革，切实保障被征地农民合法利益。深入开展农村商品流通改革和市场建设试点工作，全面完成“万村千乡”市场工程建设，加快建设“新网工程”，努力构建与新农村建设相适应的市场流通模式。 
    （四）实施重点领域突破，全面提升服务业发展水平 
    培植壮大旅游业。把今年定为“旅游发展年”，进一步创新旅游业发展机制，强化旅游整体规划，整合旅游资源，推进重点旅游项目建设，塑造和提升城市旅游品牌，营造优秀旅游城市新形象。大力推进采石风景区的综合开发，使之早日成为5A级旅游风景区。培育濮塘风景区特色休闲旅游产品。推进青山、横山风景旅游区项目建设，提高旅游与文化的融合度。积极开发生态农业观光旅游，着力发展“农家乐”，实现乡村旅游发展新突破。大力开发特色旅游纪念品，增强旅游景点的吸引力。进一步加大旅游宣传促销力度，促进区域旅游合作，全力打造区域性旅游目的地城市。 
    大力拓展物流商贸业。认真实施物流及商业网点布局规划，发挥综合交通优势，依托各类开发区和工业集中区，加速做大做强现代物流业。推进花山商贸旅游园、生产资料调剂市场建设，完善安民农副产品批发市场功能。筹建区域性钢材大市场。发展和完善特色商业街区，建成沃尔玛、华润苏果等大型超市，建设上海联华、百安居超市、滨江国际中心和红东餐饮街，打造大华国际购物广场商业核心区，提升解放路商业街、花山建材街服务功能，整合改造湖西路步行街，进一步改善消费环境。 
    努力提升新兴服务业。着力营造良好的金融生态环境，继续推动金融业改革发展，做大做强地方金融机构，提高金融服务现代化水平。支持交通银行马鞍山分行挂牌运营，实施农合行与世界银行合作对微小企业贷款项目。大力发展保险业务，开发新型保险险种，使保险业成为金融发展的新亮点。加快信息基础设施建设，积极发展电子政务、电子商务，推进企业信息化、社会信息化工作，促进信息技术在经济社会各领域的广泛应用。拓展社区便民利民服务领域，增强服务功能，提升服务水平，满足居民多样化的需求。积极培育行业协会，引导中介组织规范化发展。 
    （五）进一步加强城市规划建设管理，完善城市综合服务功能 
    切实加强规划的编制管理。依据城市总体规划和近期建设规划，加快各类专项规划和控制性详规编制工作，使控制性详规覆盖主副城区。编制完成城市紫、绿、黄、蓝“四线”规划，编制实施《主城区景观风貌保护和塑造规划》。结合正在实施的一批重大基础设施建设，进一步开展规划前瞻性研究，为全面接轨长三角、共建马芜铜宜城市群创造更为有利的条件。继续强化规划监督和管理，确保各类规划全面落实。 
    继续推进基础设施建设。坚持城市道路新建与改造相结合，重点建设路网衔接完善体系，整治改造二级道路，进一步增强城市路网功能。续建江东大道、印山路、霍里山大道，改造天门大道中段，延伸湖东南路，贯通东湖路、康乐路。实施马向路东段市政化改造，帮助和支持向山镇加快发展。继续开展城市出入口整治，加强城市基本功能配套建设。采取“以奖代补”的方式，鼓励三区实施城区二级道路和老旧小区维修改造。加快市政公园建设步伐，启动建设东湖公园等园林工程，提升城市园林绿化水平。建成王家山污水处理厂改扩建工程，推进采石河综合整治，启动慈湖河与黄家塘清淤、疏浚、分段改造工程。加快码头扩建改造步伐，实施500千伏马鞍山开关站扩建主变工程。强力推进马鞍山长江大桥、芜申运河当涂段建设，启动东环路高速化改造，争取开工建设城际铁路，逐步完善城市综合交通网络。贯彻落实房地产市场宏观调控政策，优化房地产市场环境，改善住房供应结构，全年竣工房屋面积180万平方米，提高住房保障能力。 
    进一步提升城市管理水平。巩固扩大“城市管理年”活动成果，推动城市管理向纵深延伸。坚决遏制城区新增违法建设，将拆违向城中村、城乡结合部和农村集体土地拓展，建立拆违整治示范街区。继续集中整治占道经营，推进渣土运输专项整治，有效治理城市“牛皮癣”顽症。不断加大环卫管理力度，推行“四位一体”管理方式，强化“门前三包”工作，形成高效率的保洁作业网络。高标准建设一批环卫基础设施，重点加强城乡结合部环境卫生管理，推进社区物业管理。进一步健全城市管理长效机制，全面实施城市网格化管理，实现城市管理模式根本性转变。继续深化城乡户籍制度、住房制度等一系列改革，加快农村人口城市化进程，确保全年城市化率提高2个百分点。 
    （六）积极推动全民创业，加速发展民营经济 
    全面落实促进全民创业的政策措施。把支持创业作为致富百姓的首要途径，继续广泛开展创业宣传，加大政策措施落实力度，推动全民创业、自主创业、艰苦创业，不断增加群众的经营性、财产性收入。加快建设创业园、创业街等各类创业载体，全年新建创业基地10万平方米以上，为创业者提供适用、廉价的创业场所。深入推进银政企合作，建立银行服务全民创业的考核体系和责任机制，促进各类担保公司加快发展，进一步解决小企业和各类创业主体融资难问题。完善培训服务体系，改进培训方式，加大培训力度，力求各类创业人员都能得到培训资助。建立创业项目征集、评估服务体系，完善民营企业法律服务中心功能。支持工会、共青团、妇联等群团组织开展扶持创岗创业活动，努力为全民创业提供全方位、多层次服务。 
    加大对民营经济发展的扶持力度。坚持把发展民营经济作为调整经济结构、增强经济活力的重要举措，贯彻落实鼓励发展民营经济的各项政策措施，引导民营经济快速发展。大力营造各类所有制企业公平竞争的市场环境，鼓励民间资本进入金融服务、基础设施、公用事业、社会事业等领域，使民营经济成为经济增长的重要力量。推动民营企业加快管理创新，帮助民营企业加强质量、计量、研发、检验、标准化等技术基础工作，全面提升民营企业管理水平。引导民营企业增强法制观念、诚信意识和服务社会的意识，促进民营企业健康发展。力争全年新发展民营企业1500户以上。 
    （七）加大科教兴市战略实施力度，加快建设创新型城市 
    提高企业自主创新能力。实施技术创新引导工程，鼓励优势企业申报成为国家、省级创新型试点企业，加快马钢、华菱、山鹰等重点企业自主创新步伐。加大名牌战略实施力度。鼓励企业采用高新技术、信息化技术和先进适用技术改造提升传统产业。加强对民营科技企业的扶持，加快培植一批拥有自主知识产权、具有竞争优势的高新技术企业。深入推进国家“863”新材料产业化基地主体园区建设，发挥特色园区的示范带动作用，着力壮大新材料产业。以马鞍山经济开发区为平台，加快引进和培育电子信息、节能环保、生物医药产业，争取成为省级高新技术产业开发区。力争全市高新技术产业产值、技工贸总收入双双突破200亿元。 
    建立健全科技创新体系。认真落实促进科技创新的各项政策措施，健全以企业投入为主体、社会各方广泛参与的科技创新投入体系。重点建设两个国家级工程技术研究中心，组织实施一批重大产业技术研发、重大装备技术研制项目，支持相关企业加快国家和省级技术中心、工程技术研究中心建设。按照国家级创业中心的标准，加快科创中心建设步伐，基本建成后续工程软件园和配套服务楼。鼓励企业与高校、科研院所开展多方位合作，加速产学研一体化进程。实施当涂县科技富民强县专项行动计划，推进花山区省级民营科技园、博望镇国家星火技术密集区建设，进一步增强科技对县区经济社会发展的支撑力。 
    加快教育现代化步伐。坚持教育优先发展，夯实人才培养和科技创新的基础。全面实施素质教育，推动基础教育均衡发展。实行农村义务教育“两免一补”，开展农村中小学标准化建设试点，启动农村寄宿制学校建设，加强留守儿童教育与管理。推进高中教育优质化战略，加快发展农村高中阶段教育，使全市高中阶段教育毛入学率提高到73%以上。大力发展以就业为导向的各类职业教育，加快建设当涂县职教中心，增强技能型人才培养能力。完成安工大职业技术学院迁建、河海大学闻天学院（筹）一期工程，推进安工大东校区二期工程建设，扩建改造一批中小学。积极发展民办教育，促进中外合作办学。加大人才培养、引进和使用力度，进一步改善人才创业环境。 
    三、加速构建和谐马鞍山 
    把和谐社会建设摆在更加突出位置，以解决人民群众最关心、最直接、最现实的利益问题为重点，立足当前、着眼长远，量力而行、尽力而为，努力提高人民群众的满意度和幸福感。 
    （一）统筹城乡就业，发展和谐劳动关系。把扩大就业和再就业作为经济社会发展的优先目标，以全民创业促就业为主线，扎实开展全国统筹城乡就业试点工作。建设当涂县农村劳动力专业市场，加强乡镇劳动保障工作平台和信息网络建设，规范街道劳务公司运作，扩大村级劳务公司设立范围，大力拓展劳务输出。开展“促进充分就业社区”创建活动，强化对城乡就业困难群体的帮扶，加快消除城镇“零就业”家庭。建立城乡一体化的培训体系，实施下岗失业人员再就业、农村富余劳动力转移就业和创业三大培训工程，着力提高城乡劳动者择业技能。实施劳动合同“三年行动计划”，全面推行劳动用工登记制度，深入推进集体合同制度，进一步规范劳动用工管理。健全劳动保障监察体制和劳动争议调处仲裁机制，严格劳动保障监察执法，综合治理拖欠农民工工资问题，切实维护劳动者合法权益。全年新增就业岗位2.1万个，其中失业人员再就业1万人，“4050”人员再就业2000人；开展职业技能培训1万人。 
    （二）健全城乡社会保障制度，提高人民群众生活水平。以民营企业、城镇个体工商户和农民工为重点，进一步扩大基本养老、失业、医疗保险覆盖范围，强化社会保险费征缴。完善养老金计发办法，扩大企业年金制度，形成多层次的养老保险体系。开展养老保险个人账户做实工作，完善养老保险市级统筹；切实搞好被征地农民基本养老保??老保险准备工作。加强对失业保险金发放工作的管理，健全失业保险与促进就业联动机制。巩固扩大新型农村合作医疗保障和市区城镇非职工居民基本医疗保险成果，实施当涂县城镇非职工居民基本医疗保险制度，实现医保全覆盖。加快落实农民工参加工伤保险“平安计划”。完成农村低保扩面，实现全市农村低保全覆盖。继续完善困难群众生活、医疗、子女就学等救助制度，加大对困难群体的帮扶力度。大力推进危旧房改造，全面完成本届政府确定的50万平方米的改造任务。进一步完善住房保障制度，逐步解决低收入家庭住房困难。努力扩大住房公积金实施面，推进非公企业建立公积金制度。积极推进企业退休人员移交社区管理，做好孤寡和异地居住退休人员服务工作。大力发展老龄事业，依托社区开展居家养老服务社会化示范创建活动。加快农村敬老院改扩建步伐，进一步完善和落实农村五保供养政策。出台残疾人困难家庭帮扶办法，保障残疾人合法权益。倡导奉献爱心，发展慈善事业。 
    （三）加强环境保护，推进生态市建设。树立环境立市的理念，以解决危害群众健康和影响可持续发展的环境问题为重点，加快建设资源节约型、环境友好型社会，促进人与自然和谐相处。落实循环经济发展规划，开展循环经济试点工作，争取马钢、山鹰进入国家循环经济试点单位行列。加强对马钢和马鞍山经济开发区、慈湖经济开发区内重点企业的管理，实现资源高效利用，提高资源产出效益。将节能降耗纳入县区、开发区和重点耗能企业考核目标，确保完成节能降耗年度任务。依法严格保护耕地，珍惜和合理利用土地资源，大力实施土地整理开发，提高土地集约利用水平。全面实施生态市建设总体规划。推进环境综合治理，抓好污染减排工作，重点保障饮用水安全。继续实施重点领域生态工程建设，加强矿区生态恢复，广泛开展生态创建活动。加快林业生态建设，实施马芜高速和马濮旅游大道绿色长廊、新农村林业生态示范村镇绿化等十大重点工程，不断丰富生态城市内涵，力争进入全国绿化模范城市行列。 
    （四）协调发展社会事业，推动社会全面进步。以建设和谐文化为着力点，深化文化体制改革，实施文化建设规划，加快发展文化事业和文化产业。充分发挥地方文化特色，加大李白文化品牌产品的开发力度，继续办好中国李白诗歌节。基本建成博物馆、新图书馆，加快建设大剧院。加强县区文化设施建设，广泛开展文化进农村、进社区、进企业、进校园活动，加速构建公共文化服务体系。加强文化遗产的保护和利用，规划佳山乡烟墩山遗址公园，制定当涂民歌保护规划。强化专业艺术人才队伍建设，创作更多的文化精品。进一步加强突发公共卫生事件应急救治处理能力建设，健全区、乡镇基层公共卫生服务网络。积极推进城市医疗服务体系改革，引导社会资源投资医疗服务领域。加强乡镇卫生院建设，大力发展社区卫生服务。优化城乡卫生资源配置，建立覆盖城乡居民的基本卫生保健制度。继续开展整顿和规范食品药品市场专项行动，保障人民群众饮食用药安全。启动建设市体育中心，举办市第九届运动会，广泛开展全民健身运动。筹建新广播电视中心，加快城市有线数字电视整体转换进程。稳定低生育水平，综合治理出生人口性别比偏高问题，切实提高出生人口素质。搞好第二次全国农业普查。争取加快建成马鞍山国家一级气象站。加强对国家重点档案的抢救、保护和利用，完成《马鞍山市志》续志工作。 
    （五）加强精神文明和民主法制建设，维护社会稳定。把社会主义核心价值体系融入精神文明建设的全过程，构筑全市人民团结和睦、共同奋斗的思想道德基础。践行社会主义荣辱观，深入开展社会公德、职业道德、家庭美德教育。以政务诚信、商务诚信、社会诚信建设为重点，不断增强全社会诚实守信意识，加快“信用马鞍山”建设。加强精神文明创建工作，争取跨入全省首批文明城市行列。强化民族宗教事务管理，促进民族和谐、宗教和顺。进一步搞好基层民主政治建设，以城市社区第三届换届选举为契机，探索和加强社区居民自治，加快特色和谐社区建设进程。集中开展限制养犬、限制燃放烟花爆竹专项整治活动，倡导社会文明新风尚。全面实施“五五”普法，加快形成公民自觉学法守法用法的氛围。拓展和规范律师、公证法律服务，加强和改进法律援助工作，促进社会公平和正义。大力推进“平安马鞍山”建设。组建公安特警队，实施社区警务和农村警务战略，进一步完善社会治安防控体系。依法严厉打击各种刑事犯罪，强化治安突出问题集中整治，力争早日进入全国社会治安最佳城市行列。加强对未成年人的教育和保护，预防青少年违法犯罪。全面推行社区矫正工作，落实刑释解教人员帮教任务。强化信访工作责任制，加大矛盾纠纷排查调处力度，拓宽群众利益表达与诉求渠道，最大限度地减少不和谐因素。全面落实安全生产责任制和责任追究制，深入开展非煤矿山、危险化学品、交通运输、建筑施工及消防安全等专项整治，坚决遏制重特大事故发生。加强政府应急管理体系建设，加大公共安全科技和基础设施投入，抓好预案演练和应急救援队伍建设，提高应对突发公共事件的能力。加快推进人防设施建设，进一步做好人防工作。完善国防动员体制机制，适时开展动员演练，不断提升国防动员质量与水平。贯彻落实协调发展方针，加紧建设市民兵综合训练基地，推动民兵预备役建设又好又快发展。深入开展全民国防教育，广泛开展双拥共建活动，积极创建“全国双拥模范城市”。 
    四、大力推进政府自身建设和管理创新 
    全市政府系统承担着推动经济社会跨越式发展的历史重任，必须进一步加强自身建设，大力推进管理创新，全面提高科学行政、民主行政、依法行政的能力和水平。 
    （一）进一步转变政府职能。继续推进政企、政事、政资分开，将更多的精力放到为市场主体创造良好的发展环境上来，放到为人民群众营造和谐的社会环境上来。进一步理顺和规范部门之间的职能关系，整合行政资源，精简人员和机构，完善政府分级管理体制。深化行政审批制度改革，进一步规范行政许可实施主体，对现行行政审批项目实行动态管理，保留的行政许可项目整体进驻行政服务中心办理。全面推行首席代表制和“一章一书”授权委托审批制度，不断完善并联审批、全程代理、三级联动等审批模式，拓宽网上审批范围。深入开展行政事业性收费清理，对保留的收费项目加强监管。深化财税体制改革，完善公共财政体制，切实做好增值税转型试点等新一轮税制改革工作。认真清理和界定非税收入管理范围，推动政府非税收入征缴网络信息化改革。依法管理和规范社会组织，鼓励社会力量办事业。 
    （二）进一步提高依法行政水平。认真贯彻各级人民代表大会常务委员会监督法和中央及省、市关于加强人民政协工作的意见，坚持向人大报告工作、向政协通报情况制度，主动接受人大及其常委会的法律监督、工作监督和政协的民主监督。继续办好人大代表议案、建议和市人大常委会审议意见以及政协提案，积极推进政府部门与各民主党派、工商联的政治协商和对口联系工作，建立定期向工会、共青团、妇联、科协等群团组织通报政府工作制度，广泛听取社会各界意见。深入推进政务公开，重点公开城乡发展规划、财政预决算报告、重大项目的审批和实施等情况，及时向社会公示行政决策的过程和结果，增强政府工作的透明度。加强对安全生产、市场监管、行政执法等责任追究制度落实情况的督查，严肃查处行政不作为和行政乱作为。分解落实行政执法职责，建立执法过错追究制，健全行政争议解决机制，进一步完善行政执法评议考核机制，规范行政执法行为。着力加强基层依法行政工作，全面增强政府的执行力和公信力。 
    （三）进一步提升行政服务效能。按照建立反应灵敏、运行快捷、协调高效的行政运行机制的要求，扎实推进服务型政府建设。认真落实部门行政首长问责制，严格执行效能建设六项工作制度，促进各级行政机关高效行政。深化、完善和落实工作流程再造，及时纠正执行过程中出现的偏差。继续开展机关作风评议，健全效能工作群众评价和监督机制，提高政府部门、窗口行业的办事效率和服务质量。加强政府工作标准化、规范化建设，努力降低行政成本。根据率先建成全面小康社会的要求，坚持“注重实绩、突出一线”的原则，完善以政府绩效为导向的目标管理体系，加大考核奖惩力度，以高效务实的工作业绩取信于民。各级政府及其部门尤其要强化全局意识、服务意识、责任意识和协作意识，坚决杜绝推诿扯皮、各行其是的现象，切实解决有人出题、无人作答的弊端。 
    （四）进一步加强公务员队伍建设。深入学习贯彻《公务员法》，加强对公务员的教育和培训，全面提高公务员队伍的政治素质、理论素质、业务素质和心理素质，不断增强工作谋划能力、解决问题能力、创新创造能力，特别是推动率先建成全面小康社会的能力。各级政府工作人员特别是领导干部，要切实增强群众观念和公仆意识，坚持求真务实，勇于开拓创新，真正把服务人民的要求落实到各项决策和行动当中。深入开展商业贿赂专项治理工作，建立健全防治商业贿赂的长效机制。继续加大政风建设力度，坚决纠正群众反映突出的不正之风。强化行政监察职能和审计监督职能，逐步推行效益审计。深入推进领导干部职务消费改革，规范公务员津补贴发放和机关公务接待。认真执行廉政建设责任制，大力推进惩防腐败体系的构建??力建设人民群众满意的政府。 
    各位代表，新的一年充满着新的希望和挑战，率先发展的使命激励我们勇往直前，人民群众的重托不容我们丝毫懈怠。让我们高举邓小平理论和“三个代表”重要思想伟大旗帜，以科学发展观为统领，紧密团结在以胡锦涛同志为总书记的党中央周围，在省委、省政府和市委的领导下，依靠全市人民，凝聚各方力量，攻坚克难、开拓进取，全面完成本届政府的各项目标任务，为加快建成全面小康社会、以优异成绩迎接党的十七大胜利召开而努力奋斗！
